# Identifying the zero-dose and under-immunized children in Bangladesh: Approaches and experiences

**DOI:** 10.1371/journal.pone.0312171

**Published:** 2024-10-28

**Authors:** Zerin Jannat, Hemel Das, Md. Wazed Ali, Nurul Alam, Mahbub E. Elahi Khan Chowdhury, Bidhan Krishna Sarker, Md. Mahbubur Rahman, Shehrin Shaila Mahmood, Md. Musfikur Rahman, Christopher Morgan, Elizabeth Oliveras, Gustavo Caetano Correa, Heidi W. Reynolds, Tasnuva Wahed, Md. Jasim Uddin

**Affiliations:** 1 Health Systems and Population Studies Division, icddr,b, Dhaka, Bangladesh; 2 Maternal and Child Health Division, icddr,b, Dhaka, Bangladesh; 3 Technical Leadership and Innovation, Jhpiego, The Johns Hopkins Affiliate, Baltimore, MD, United States of America; 4 Measurement, Evaluation and Learning (MEL), Gavi, The Vaccine Alliance, Dhaka, Bangladesh; College of Medical Sciences, NEPAL

## Abstract

This paper presents and elaborates on empirical methods and approaches used to identify Zero-Dose (ZD) and Under-immunized (UI) children as well as the communities that these children reside in within Bangladesh. This paper also describes demand- and supply side-barriers that lead to children being ZD and UI in the country. Time period for the study was December 2022—May 2023. The study methodology encompassed secondary data analysis using data from national surveys, primary data collection and analysis via a lot quality assurance sampling (LQAS) survey and also, qualitative data collection and analysis. Study population included caregivers of children aged 4.5 months (4 months 15 days) to 23 months for the LQAS survey. The qualitative component included policymakers, program managers and service providers working in immunization as well as mothers in the selected study areas who had a living child aged less than 2 years as the study population. Our data analysis confirms existence of ZD and UI children in areas which were categorized into haor (wetlands), hilly, char (sandy/silty land surrounded by water), coastal, plain land and urban slums. Determinant analysis showed that the mother’s level of education, antenatal visits made, and access to media were significantly associated with children being ZD or UI. Reproductive autonomy emerged as a key factor that had prominent impact on a child being ZD. The qualitative analysis indicated the importance of population migration, health workforce shortages and lack of access to transportation as prominent barriers to immunization. Notably, the methods and approaches used in this study are both effective and easily replicable to identify ZD and UI children. The drivers of ZD and UI along with the barriers to immunization provide potential areas for intervention by policy-makers and can apprise about interventions to be tested in future implementation research.

## Introduction

Since 1974, the Expanded Programme on Immunization (EPI) has worked to establish immunization programmes throughout the globe [1] with the aim of ensuring that all children worldwide are able to benefit from life-saving vaccines [2]. The EPI has successfully increased global vaccination coverage, leading to substantial declines in child morbidity and mortality from vaccine preventable diseases (VPD) [3,4] and saving countless lives. Every nation in the world has a national immunization programme [2,5]. In lower-income countries, 81% of children now receive routine EPI vaccines [6]. Nevertheless, extending routine vaccination coverage to all children and communities, especially in many low- and middle-income countries (LMICs), has proven to be a demanding challenge with a global estimate of 25 million children being left un- or under- vaccinated [7,8].

In simple terms, unvaccinated children are those who have not received any routine vaccine and under-immunized are those who have missed at least one recommended vaccination. To align measurement efforts, allocate resources and define success, zero-dose (ZD) children are more closely defined as children who did not receive the first dose of diphtheria-tetanus-pertussis containing vaccine (DTP1) and under-immunized (UI) children are defined as those who did not receive the third dose of DTP containing vaccine (DTP3), usually measured at age 12 months or older [9,10]. In countries like Bangladesh, where DTP has been replaced by Pentavalent vaccine, which includes antigens against DTP as well as hepatitis B and *Haemophilus influenzae* type b, ZD children may be defined as those who did not receive the first dose of pentavalent vaccine (PENTA-1) and those who missed the third dose of pentavalent vaccine (PENTA-3) may be considered UI. We followed these definitions of ZD and UI for our research.

It is estimated that one out of every five children in the world is ZD or UI and therefore, susceptible to virulent VPD [8]. Approximately, 50% of ZD children reside in three geographical contexts: informal or peri-urban, remote-rural, and conflict areas [6]. Despite growing efforts over the past decade to expand immunization, the number of ZD children declined by only 4.1% from 2000–2021 [8] and the COVID-19 pandemic led to severe disruptions in access to routine childhood vaccination. UNICEF estimates that between 2019 and 2021, 67 million children missed at least one routine immunization and about 48 million of these children were ZD [8].

Since its establishment in 2000, Gavi, the Vaccine Alliance, has supported low-income countries to introduce new vaccines and vaccinate over one billion children worldwide [10]. Recently, Gavi, under its Gavi 5.0 strategy and the global Immunization Agenda 2030 have focused on immunization equity or reaching ZD and UI children and their communities. Gavi set up Country Learning Hubs (CLHs) in Bangladesh, Mali, Nigeria, and Uganda with the aim of using evidence to understand the factors influencing implementation and performance of approaches to identify and reach ZD and UI children.

In Bangladesh, where the highly successful EPI program resulted in an increase in full vaccination coverage from less than 2% in 1985 to 83.9% in 2019, communities remain where as many as 15% of children are un- or under-vaccinated [11]. Identifying and addressing these pockets of ZD and UI children is critical to improving immunization coverage and ensuring population protection against VPD. The Bangladesh CLH is tasked with developing the evidence base for tools and approaches that can be used to reach these children and their communities. This paper describes the results of the preliminary activities of the Bangladesh CLH to identify ZD and UI children and detect missed communities—both the methods used and the results—as part of a rapid assessment.

## Methods and approaches

Between December 2022 and May 2023, we collected and analyzed both primary and secondary data to identify potential missed communities and barriers to immunization as the first step in an implementation research study. We identified missed communities using a three-step process: 1) identification of high prevalence of ZD upazilas (i.e. sub-districts), 2) identification of potential pockets of ZD within the selected upazilas, and 3) conduct lot quality assurance sampling (LQAS) survey to confirm whether or not the pockets were high prevalence. We conducted qualitative mapping to describe the characteristics of the missed communities, quantitative analysis of secondary data to identify factors related to ZD and UI, and interviews with service providers and mothers of children to identify barriers to immunization. Each method is described below.

### Identifying missed communities

#### Sampling methods

In step 1, we analyzed data from the Bangladesh Coverage Evaluation Surveys (CES) conducted in 2014, 2015, 2016 and 2019 [11–14] to identify high ZD prevalence districts and city corporations (CCs). Our primary outcome was percentage of children aged 4.5–23 months who had not received the first dose of pentavalent vaccine (PENTA-1) and our secondary outcome was the percentage of eligible children who had not received the third dose of pentavalent vaccine (PENTA-3). This age range was selected to ensure that the youngest children were eligible for Penta-1 vaccination, including the grace period commonly allowed in Bangladesh, and that the oldest children were eligible to have received Penta-3. We sorted the districts and CCs in descending order according to the percentage of ZD children for each year and ranked the highest 10 districts and 5 CCs. To identify upazilas in rural districts, we ranked upazilas by the percentage of ZD children reported in the national health management information system (HMIS) from 2019 to 2022 [15]. We verified these findings using routine monitoring data capture in EPI monthly reports (Jan-Nov 2022) and EPI micro-plans that were collected from the upazila offices. We assumed that the physical reports were more accurate than the health management information system (HMIS) data and updated the percentage of ZD children in the national HMIS database (DHIS2) using these reports when there was a discrepancy. For the CCs, we verified high ZD zones using routine data from EPI headquarter (HQ) for the year 2022 [Jan-Nov].

In step 2, to identify pockets of ZD/UI within the identified upazilas we consulted the EPI service providers of the selected upazilas and zones to first identify unions (lower level administrative units) likely to have high numbers of ZD or UI children. Within those unions, we used input from service providers and administrative reports to identify two EPI clusters (i.e., catchment area of a vaccination center) within each union likely to have the highest rate of ZD or UI children.

In step 3, we used LQAS to confirm whether or not the selected clusters were missed communities (had a high prevalence of ZD). Details about the LQAS is described below:

#### Lot Quality Assurance Sampling (LQAS)

The LQAS involved primary data collection from respondents. Through LQAS sampling process, we incorporated a quantitative approach to identify a missed community based on small sample size and set a decision value based on ZD or UI status for accepting or rejecting a cluster. The primary data collection commenced on March 23, 2023 and ended on April 30, 2023. Written informed consent was obtained from each respondent.

Caregivers of children aged 4.5 months (4 months 15 days) to 23 months were interviewed for LQAS survey. The inclusion criteria of caregivers included respondents aged above 18 years who were taking care of at least one child aged between 4.5–23 months. They were usual residents (staying at least for 3 months) of the households in our selected study clusters.

#### Sample size determination for LQAS

According to Bangladesh CES 2019, the valid PENTA-3 vaccination coverage is 93.3% [16]. This implies that about 7% children are either ZD or UI at the national level with geographic variation. We applied the LQAS method under single sampling plan to select clusters with high (≥ 10%) ZD or UI for this study. We assumed that the prevalence of ZD or UI children follows the binomial probability distribution and limited the type I error to 5%. We set the decision value to *d* = 5. According to Sathakatullah and Murthy [17], to detect the clusters with ZD or UI prevalence *P*≥0.10 and *d* = 5, the required sample size of households with eligible child in each cluster is 28. Thus, we conducted 28 interviews from each cluster.

#### Sampling design for LQAS

As part of the rapid assessment, we used LQAS to confirm the clusters with a high percentage (>10%) of ZD or UI children according to the secondary survey and DHIS2 data analyses are under immunized clusters. LQAS is a quantitative approach based on small number, but the results are/should only be interpreted qualitatively at the lot/cluster level and used as strong evidence to make conservative inference to protect any errors due to small sample sizes typically used in LQAS. As the conservative null hypothesis is P (prevalence of ZD and UI) ≤10%, meaning any value ranging between 0 and 10. LQAS was planned to conduct in 24 clusters in 12 upazilas (sub-districts)/zones. The confidence interval and the power of the test was calculated from pool data.

According to sampling design, we set a decision value for cluster to accept or reject it based on ZD or UI status. If any cluster had higher number of ZD or UI children than the decision value, it was identified as a missed community. The details in this regards are described below:

#### Selection of cluster for LQAS

Stated prior, a total of 10 upazilas and 2 zones of Dhaka North CC were preliminarily selected based on the results of secondary data analysis that showed they had a high proportion of ZD or UI children. The EPI service providers of the selected upazilas and zones were consulted to identify unions (lower level administrative unit) likely to have high numbers of ZD or UI children from the selected upazilas. After that, two EPI clusters with highest rate of ZD or UI children within the union of the upazila or zone were selected. The selected EPI clusters in the upazilas was classified into six different geographic locations—haor, hilly, coastal, plain land, char and urban slum.

#### Data collection for LQAS

A total of six field research assistants (FRAs) with experience in survey data collection were recruited. They were trained on the LQAS process. They were instructed to visit the EPI center of the enlisted clusters. The FRAs commenced their data collection from the household situated in the north-east corner of the selected cluster and searched for children who were born between March 25, 2021 and November 08, 2022. The reasons for following purposive sampling method were unavailability of the list of households with children aged 4 months 15 days to 23 months 29 days and selecting north-east corner of the selected EPI outreach center (cluster) to avoid the possible selection bias by data collectors. The data collectors collected information from caregivers of the child from the eligible household. They visited then the next door until 28 households were interviewed. All the clusters did not have 28 eligible households. In such cases, the interviewer went to the nearest adjacent EPI cluster to complete the required sample size. The questionnaire was pre-tested before data collection.

#### Ethical consideration

We obtained approval from the Research Review Committee (RRC) and Ethical Review Committee (ERC) of Institutional Review Board (IRB) of International Centre for Diarrhoeal Disease Research, Bangladesh (icddr,b) for this study prior to data collection as well as implementation. All participants were protected when invited to take part in the study and respondents were interviewed only after their written informed consent was obtained. Participation was voluntary and the participants were ensured that their refusal would have no adverse consequences for them. All data collected were kept confidential and the team took all necessary steps to protect the privacy of personal information.

### Exploring characteristics of missed communities, factors related to ZD and UI, and barriers to immunization

Once the missed communities were confirmed using LQAS, the data collectors did a transect walk of the cluster and drew a physical map of the area based on their observations and informal discussions with community members. They mapped characteristics of the locality, including landmarks, population type, and household structures.

#### Data analysis

For exploring the factors related to ZD and UI, we performed secondary data analysis. We used a multivariate logistic regression analysis as a statistical tool utilizing dataset from the 2017–2018 Bangladesh Demographic and Health Survey (BDHS) to identify socio-economic determinants of two binary outcomes- ZD and UI. We then conducted a determinants analysis to discern socio-economic determinants for children being ZD or UI; using a listing of confounders derived from our experience and published literature. We calculated both crude and adjusted odds ratio (AOR) for the outcomes with respect to the determinants to evaluate the pattern of association between the outcomes and factors. We interviewed key informants including national and sub-national EPI managers and service providers (n = 28) and caregivers of eligible children (n = 10) to identify demand and supply side barriers to immunization and triangulate the quantitative results.

## Results

Identifying Missed Communities using CES and Routine Service Delivery Statistics depicts the results of our secondary data analysis (Table 1). At the initial stage of identification of missed communities, we found that Jamalganj upazila of Sunamganj district at Sylhet division had the highest ZD prevalence (11.1%). However, when we verified our initial findings and updated the data as described in the above section, we found that Gaffargaon Upazila of Mymensingh at Mymensingh division, in fact, had the highest prevalence (15.7%) with Jamalganj upazila placing second in regards to ZD prevalence. The top 30 upazilas were located across 21 different districts in all eight divisions. The average estimated prevalence of ZD was lower in the top highest prevalence zones in CCs at just over 1% compared to the average of 4% found in the 21 highest prevlance districts. This may be because urban areas have more socioeconomic diversity and this mutes the effects of ZD at the population level, even though there are pockets that are higher ZD prevalence.

**Table 1:** Top high ZD upazilas identified after updating routine EPI data.

Sl.	Division	District	Upazila	ZD (%)
1.	Mymensingh	Mymensingh	Gaffargaon Upazila	15.7%
2.	Sylhet	Sunamganj	Jamalganj Upazila	11.1%
3.	Barisal	Jhalokati	Kathalia Upazila	10.8%
4.	Khulna	Meherpur	Mujibnagar Upazila	10.2%
5.	Rajshahi	Chapai N.	Shibganj Upazila	9.9%
6.	Chattogram	Bandarban	Ruma Upazila	9.2%
7.	Rangpur	Gaibandha	Saghata Upazila	8.5%
8.	Khulna	Bagerhat	Sadar Upazila	8.2%
9.	Dhaka	Manikganj	Ghior Upazila	7.5%
10.	Rajshahi	Chapai Nababganj	Sadar Upazila	7.4%
11.	Chattogram	Cumilla	Debidwar Upazila	7.4%
12.	Rajshahi	Joypurhat	Akkelpur Upazila	6.6%
13.	Rajshahi	Joypurhat	Sadar Upazila	6.4%
14.	Chattogram	Chattogram	Chandanaish Upazila	6.3%
15.	Sylhet	Sunamganj	Dowara Bazar Upazila	6.0%
16.	Sylhet	Sunamganj	Dharampasa Upazila	5.6%
17.	Rangpur	Gaibandha	Palashbari Upazila	5.5%
18.	Chattogram	Noakhali	Hatiya Upazila	5.4%
19.	Chattogram	Laksmipur	Ramgoti Upazila	5.2%
20.	Sylhet	Sunamganj	Sulla Upazila	5.1%
21.	Khulna	Jhenaidaha	Sadar Upazila	5.0%
22.	Mymensingh	Netrokona	Khaliajuri Upazila	4.5%
23.	Chattogram	Noakhali	Sonaimuri Upazila	4.5%
24.	Khulna	Jhenaidaha	Kaliganj Upazila	4.4%
25.	Mymensingh	Mymensingh	Sadar Upazila	4.3%
26.	Rajshahi	Sirajganj	Sadar Upazila	4.3%
27.	Sylhet	Moulavi Bazar	Rajnagar Upazila	4.2%
28.	Barisal	Patuakhali	Dashmina Upazila	4.2%
29.	Chattogram	Rangamati	Sadar Upazila	4.1%
30.	Sylhet	Sylhet	Sadar Upazila	4.1%

Data source: DHIS2 (2022).

**Fig 1:**
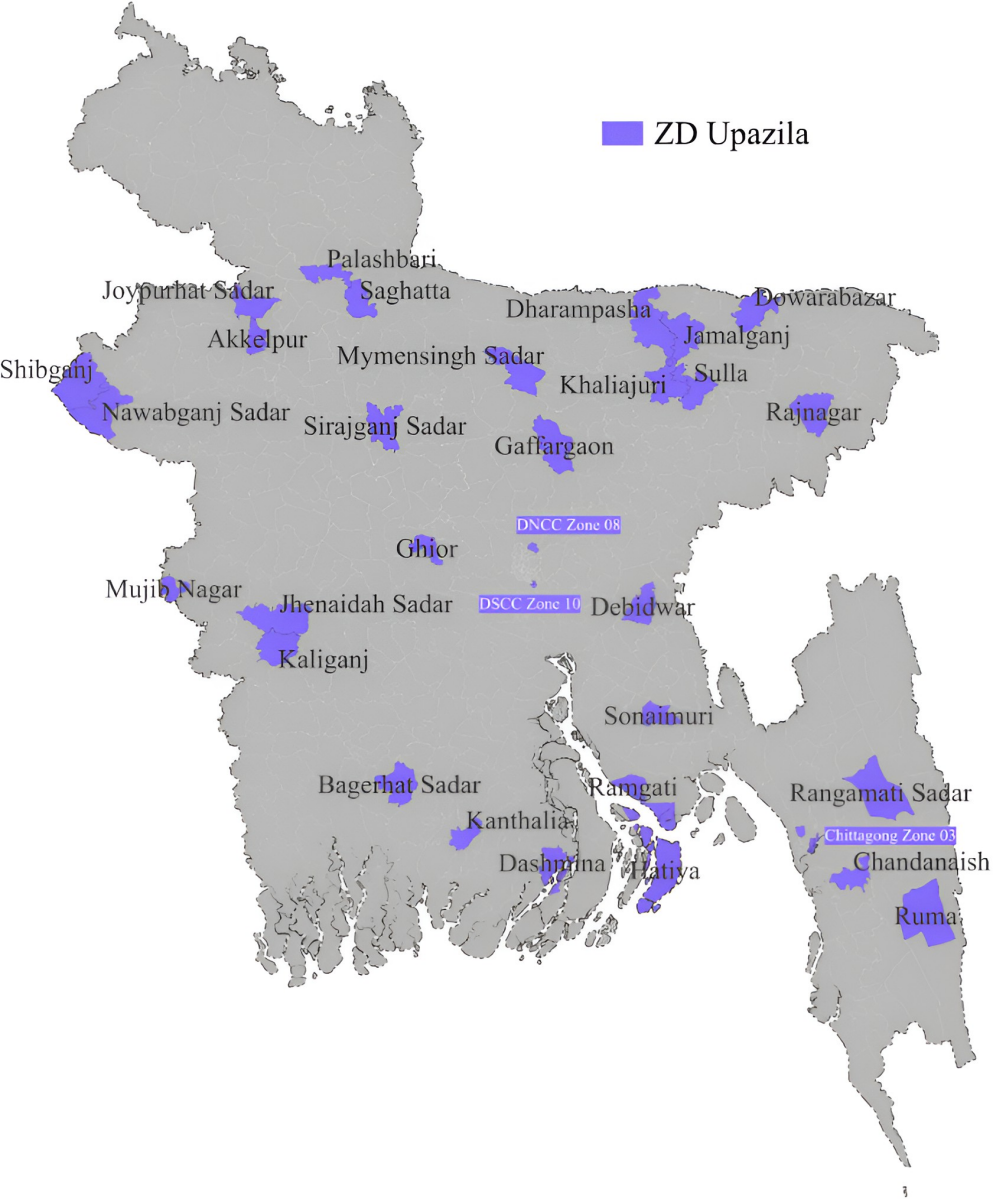
Geographic distribution of high ZD upazilas identified using Routine EPI data (2022).

This is further illustrated in Fig 1 below which pictorially shows the top high ZD upazilas we identified from our secondary data analysis.

### Socio-economic Determinants of ZD and UI

The determinant analysis found that mothers’ education, occupation, number of antenatal visits made, mother’s access to media and geographical location were associated with children being ZD or IU (Table 2). In contrast, type of residence and wealth quintile were observed not to have any significant association with children being ZD or UI.

**Table 2:** Socio-economic determinants for children being ZD and UI [BDHS 2017–18].

Covariates	ZD			UI		
	n	AOR^a^ (95% CI)	p-value	n	AOR^a^ (95% CI)	p-value
**Mother’s education**						
No education (Ref)	278	-	-	257	-	-
Primary incomplete	764	0.99 (0.55–1.77)	0.961	711	0.85 (0.57–1.28)	0.439
Primary complete	470	0.62 (0.31–1.23)	0.168	434	0.57 (0.35–0.92)	0.021
Secondary incomplete	1931	0.26 (0.13–0.51)	<0.001	1813	0.44 (0.29–0.66)	<0.001
Secondary complete or higher	1061	0.22 (0.09–0.54)	0.001	977	0.32 (0.19–0.55)	<0.001
**Mother’s occupation**						
Working (Ref)	1815	-	-	1725	-	-
Not working	2689	2.80 (1.73–4.51)	<0.001	2467	1.42 (1.10–1.83)	0.007
**Number of ANC visits**						
0 (Ref)	365	-	-	335	-	-
1–3	1941	0.53 (0.32–0.88)	0.015	1805	0.64 (0.45–0.91)	0.013
> = 4	2198	0.37 (0.21–0.68)	0.001	2052	0.45 (0.30–0.67)	<0.001
**Division**						
Rangpur (Ref)	501	-	-	474	-	-
Barisal	474	1.82 (0.56–5.88)	0.316	431	1.04 (0.56–1.94)	0.906
Chattogram	744	1.21 (0.37–3.97)	0.753	698	1.63 (0.94–2.82)	0.08
Dhaka	678	1.93 (0.62–5.99)	0.255	634	1.63 (0.92–2.88)	0.092
Khulna	465	2.24 (0.67–7.46)	0.191	436	2.20 (1.23–3.92)	0.008
Mymensingh	546	2.53 (0.82–7.77)	0.106	501	1.58 (0.90–2.77)	0.115
Rajshahi	479	0.95 (0.23–3.87)	0.943	451	1.32 (0.71–2.43)	0.381
Sylhet	617	5.02 (1.74–14.49)	0.003	567	2.40 (1.41–4.10)	0.001
**Wanted last child**						
Wanted then (Ref)	3545	-	-	3298	-	-
Wanted later	598	1.71 (1.03–2.9)	0.04	560	0.99 (0.69–1.4)	0.942
Wanted no more	361	1.21 (0.66–2.2)	0.535	334	0.96 (0.64–1.44)	0.848
**Media access**						
Yes (Ref)	2457	-	-	2295	-	-
No	2047	1.49 (0.94–2.37)	0.089	1897	1.40 (1.06–1.86)	0.019

^**a**^ adjusted for sex of child, number of ANC visits, division, type of residence, mother’s education, wealth quintile, wanted last child, mother’s occupation and media access.

The possibility of a child being ZD or UI was significantly lower if the child’s mother had more than primary education. Children of mothers who made more antenatal visits or of working mothers were less likely to be ZD and UI compared to their counterparts. We also observed that geographical location played a part, children living in Sylhet are more likely to have ZD or UI children than those residing in Rangpur.

These associations were all seen across both ZD and UI children. One factor, however, was only associated with children being ZD (and not with UI), that is: mothers who reported that “their last child was wanted” were less likely to have a ZD child. The details of the determinant analysis along with crude and adjusted odds ratio (AOR) of ZD and UI by the covariates can be found in (S1 and S2 Tables).

### Results from LQAS data analysis

Findings from the LQAS analysis confirmed the presence of missed communities across the districts identified in the secondary analysis (Table 3). A total of 18 clusters (16 from eight upazilas and two from one CC) were accepted as high either high ZD or UI based on the LQAS survey.

**Table 3:** Confirmation of ZD and UI areas using LQAS.

District	Upazila	EPI Cluster	Total	ZD	UI	Decision Value≥5	Primary selection
Sunamganj	Jamalganj	Cluster-1	28	0	5	Accept	Comparison
		Cluster-2	28	1	5	Accept	
	Dowara Bazar	Cluster-1	28	4	19	Accept	Intervention
		Cluster-2	28	6	3	Accept	
Chapai Nawabganj	Shibganj	Cluster-1	28	0	2	Reject	Dropped
		Cluster-2	28	0	1	Reject	
	Sadar	Cluster-1	5	0	0	Undecided	Dropped
Gaibandha	Saghata	Cluster-1	28	2	10	Accept	Intervention
		Cluster-2	28	1	5	Accept	
	Fulchhari	Cluster-1	28	2	8	Accept	Comparison
		Cluster-2	28	0	5	Accept	
Noakhali	Hatiya	Cluster-1	28	3	6	Accept	Intervention
		Cluster-2	28	2	4	Accept	
	Subarnachar	Cluster-1	28	5	1	Accept	Comparison
		Cluster-2	28	3	3	Accept	
Rangamati	Sadar	Cluster-1	8	0	0	Undecided	Restriction on mobility from local government authority
		Cluster-2	8	0	0	Undecided	
		Cluster-3	28	0	4	Reject	
		Cluster-4	16	0	1	Undecided	
		Cluster-5	16	0	0	Undecided	
		Cluster-6	2	0	1	Undecided	
	Naniarchar	Cluster-1	4	0	0	Undecided	
Sherpur	Nalitabari	Cluster-1	28	0	5	Accept	Intervention
		Cluster-2	28	0	7	Accept	
	Sreebardi	Cluster-1	28	0	5	Accept	Comparison
		Cluster-2	28	0	7	Accept	
Dhaka	Dhaka North CC (DNCC)	Cluster-1	28	5	12	Accept	Intervention
		Cluster-2	28	5	11	Accept	Comparison
	**Total (Accepted Cluster)**		**504**	**39**	**122**		
**Prevalence of ZD: 7.7% and UI: 24%**							

Table 1 also depicts the list from which we identified potential areas where we wanted to conduct the LQAS for final selection of study areas where we will conduct implementation research (IR) as the next step to bring the success of Bangladesh CLH to fruition. We prioritized areas with high ZD and UI for study area selection but we considered districts found to have at least two upazilas with ZD children in a district/CC. This was done to enhance the study area bifurcation into intervention and comparison upazilas for the IR. Table 3 shows the results of LQAS. In LQAS sample, the prevalence of ZD and UI among the accepted cluster were 7.7% and 24%, respectively.

We were unable to conduct initial LQAS in Rangamati due to security concerns, however, we were later able to gain support from governmental officials and decided to include Rangamati as an implementation research (IR) study site.

Overall, we prioritized ten upazilas from five districts and two zones from one CC for the implementation resrarch. These five districts and one CC reflect the variety of Bangladesh’s ecological settings: haor (wetlands), hilly, char (sandy/silty land surrounded by water), coastal, plain land and urban areas are shown in Table 4 below.

**Table 4:** Confirmed missed communities.

Geographic Location	Districts/CC	Upazila/Zone
Haor	Sunamganj	1. Jamalganj
		2. Dowarabazar
Hilly	Rangamati	1. Sadar
		2. Kawkhali
Char	Gaibandha	1. Saghata
		2. Fulchari
Coastal	Noakhali	1. Hatiya
		2. Subornachar
Plain land	Sherpur	1.Nalitabari
		2.Sreebardi
Urban (CC)	Dhaka	1. Zone-01 of DSCC
		2. Zone-06 of DNCC

Based on the LQAS survey, prime reasons for not having their child vaccinated included the child being sick, vaccination not being permitted by her husband, financial limitations and busy schedule of parents. Based on the observational mapping of the clusters, most clusters had primary schools but fewer had secondary and/or missionary educational institutions. Notably, the coastal clusters of Noakhali coastal, did not have any educational institutions. With regard to healthcare facilities, none of the clusters had an NGO clinic and only one cluster from an upazila of Gaibandha (Char area) and one cluster from upazila of Noakhali district had public/government health centers. Private clinics were only found at the urban clusters. Bus was the transport system only in the urban zones while boats were a prominent mode of transport used by individuals in the haor and char clusters whereas rickshaws, compressed natural gas (CNG), motorcycles and cars were used more in the coastal, plain and urban clusters. The LQAS also showed that the majority of households in almost all the clusters were mud-built (i.e. kacha), however in urban clusters households were mostly the slum shacks and mud-built followed by half-cemented residences.

### Results from qualitative data analysis

When asked about their ability to identify and reach ZD or UI children, most upazila level managers, service providers and their supervisors mentioned that they had experienced difficulties in finding or monitoring ZD and UI children. Perhaps the most important challenge not identified by the quantitative analysis was the large and severe limitations in human resources for health, especially the shortage of Health Assistants (HAs) at field level. Other factors felt to be important were inconsistent interpersonal communication (IPC) practices, and access difficulties due to road or weather conditions. In hard-to-reach (HTR) areas, interviewees mentioned that households are scattered and EPI clinics are located far from the community’s center (e.g., upazila headquarters). This combined with poor communication and transportation systems was noted to impede EPI activities here. In urban areas, on the other hand, children may live close to an EPI clinic, but the number of children is large and not clearly defined, which creates challenges for the limited workforce. Moreover, high rates of migration in slum settlements make it difficult to vaccinate and because many mother’s work as labourers, it is difficult for them to reach clinics during operating hours.

The respondents in qualitative interviews reported both demand and supply side factors that lead to ZD and UI. Notably, the factors mentioned by respondents were similar for both ZD and UI children. However, the prime demand-side factors were:

#### Migration due to environmental damage or cultural reasons

In coastal and char areas, district and upazila level health managers, including all service providers, mentioned migration due to river erosion as a key hindrance to conducting EPI activities. Migration was noted to complicate the preparation of micro plans and make tracking of ZD or UI children difficult. Migration was also mentioned frequently as a challenge in plains areas. One key informant mentioned:

"*EPI activities cannot be carried out properly in river erosion areas. Because the vaccine recipients move to other areas due to the river erosion, that means they migrate. Many people think that our area is a poor area, Char area is a poor area. Many people migrate to Dhaka for work. Due to this migration, the EPI activities are hindered."*

#### Fear of minor side effects

A few mothers and service providers mentioned that misconceptions about contraindications or concerns about side effects of vaccination sometimes hinder immunization uptake. The crying that results after vaccination as a factor that led some older family members to discourage women from bringing their children to the clinic.

#### Misconceptions and hesitancy

Only in Sunamganj district participants mentioned that religious prejudice and misperceptions about EPI were widely prevalent and affected EPI performance adversely. Notably, the Sylhet region where Sunamganj is located is as a whole, conservative (Muslim and non-Muslim) and coverage of reproductive, maternal and child health indicators are low. Some religious leaders in this area have actively spread misconceptions and even prevented EPI program staff from disseminating messages using a loudspeaker. At the same time, some participants felt that EPI providers from outside the local ethnic community were not trusted. Participants said that the misconceptions kept some families from vaccinating their children. For example, one participant said:

"*There was some social stigma in regard to vaccination, when a child get fever after taking the vaccine, the person then spread this message to other people surrounding them telling that the child got sick from receiving vaccination. This discourages other women and was a problem to carry out EPI programme in hilly areas.”*

Another informant mentioned:

"*Many mothers think that after vaccination, the child will become blind, crippled, sick etc. They think that their children won’t be alright."*

The supply-side factors that participants noted include:

#### Shortage of human resources (HR) and work overload

The respondents, across all study sites, mentioned that personnel shortages were a demanding and persistent challenge. Large catchment areas with inadequate numbers of HAs and porters were believed to lead to poor service coverage. The inadequate numbers of service providers in HTR areas has exacerbated the situation. All the HAs and their supervisors mentioned that HAs are overburdened with work. In some geographically isolated areas, the HAs work in three or more wards although officially each HA is supposed to work in just one ward. This makes visiting every site more than once a month impossible and is a prominent challenge for coverage. To exemplify, a key informant mentioned:

"*In the hard-to-reach areas, the HAs cannot move to outreach centers routinely and perform the IPC before the immunization day due to long distances, shortage of human resources, and higher transport fares."*

#### Limited opportunity to provide IPC

Across the study sites, supervisors, providers and mothers discussed limited use of IPC to inform communities in advance of outreach services. Providers noted difficulties in visiting remote sites two days in a row while some facilities managers said that even though HAs are recruited locally, they generally stay outside the area, making it difficult for them to visit the sites the day before a vaccination session.

#### Distance to EPI centers and unavailability of transport

Across all the sites, both rural and urban, travel was reported to be a substantial challenge to EPI, during both dry and rainy seasons. The service providers and service recipients struggle to cope with long distances and limited transportation.

Some HTR unions do not have any means of communication except by river, and there is no way to use any vehicles in these areas during the dry season. Hence, an HA cannot conduct EPI sessions and IPC routinely in those areas. The service providers mentioned that some EPI centers are about 45–50 km away from upazila headquarter and there is lack of transport. Some mothers and service providers said that the long time and the costs required to reach an EPI center discourages mothers form taking their children for immunization.

#### Inaccurate denominator in EPI

Almost all the national, district and upazila level participants said that they use a bottom-up approach to determining the estimated number of children eligible for vaccination (i.e., the denominator) for their micro plans, although participants described different calculations. This was more difficult in areas affected by migration as noted above. One informant said:

“*The current year’s denominator is determined by multiplying the number of children who received vaccine in the previous year by population growth rate, a method that makes calculation of the ZD burden impossible.”*

## Discussion

This study reports the methodologies and initial findings from a rapid assessment of ZD and UI children in Bangladesh conducted by the CLH. The rapid assessment is part of a larger implementation research study that will pilot targeted approaches to reaching ZD children. As well as outlining the ZD and UI challenge, these findings also help identify ZD areas that could serve as implementation research sites. Given that this was an aim of the assessment, it was not designed to find all pockets of ZD in the country, or even within the identified upazilas. However, the approach used is a potential method for identifying pockets of ZD or UI children at the local level, with some validation. The initial findings on demand- and supply- side drivers of ZD and UI are in part of specific to Bangladesh (such as ecological factors) but are also consistent with the drivers of ZD found in other settings.

Our findings demonstrate the utility of combining existing survey and administrative data for identifying missed communities, but also highlight the importance of field validation to confirm the results. This combination approach was particularly useful for ensuring that we chose the most relevant clusters of ZD children to use as intervention and comparison sites for future implementation research. That said, current surveys (e.g. CES, BDHS) are limited in their ability to provide information at the upazila and zone level needed to address ZD. However, the combination of utilizing both survey and administrative data along with application of LQAS process as monitoring tool was the innovative strategy attempted by us to identify the missed communities. Other countries also applied different methodologies to identify missed communities [18–20]. In Mali, electronic community immunization and birth registries were carried out to identify priorities and assess the burden of ZD [18]. Nigeria and Uganda conducted situational analysis and targeted surveys to identify/locate and monitor ZD children [19,20] A unique aspect of our study in Bangladesh, was the use of LQAS to confirm the presence of missed communities in the areas that secondary data analysis identified as home to ZD and UI children. The LQAS results not only confirmed the prevalence of ZD and UI in select communities in Bangladesh, but also showed that these communities exist in all eight divisions of the country and in geographically diverse areas. This supports findings from a geospatial analysis of data from 99 LMICs [7] that found ZD and UI children in remote rural, urban and peri-urban areas as well as in conflict-affected areas.

Our successful use of LQAS for confirming missed communities suggests that LQAS surveys can be used for this purpose, particularly in contexts with relatively high immunization coverage and where un- or under-coverage may be hidden in most data sets. Previously, Sandiford had suggested LQAS as an efficient method to monitor poor performance of immunization programme [21]. A recent study showed LQAS as a useful sampling technique to integrate data from different programmes in a single survey of immunization [22]. Polio Eradication & Endgame Strategic Plan 2013–2018 recommended LQAS sampling method to monitor Polio immunization method as well [23]. A study conducted in Kosovo used LQAS process to evaluate vaccination status among children aged 12–24 months [24]. We also found two studies from India that assessed full immunization coverage using LQAS sampling methodology. To the best of our knowledge, our study is the first to apply LQAS to identify the areas with ZD and UI children and successfully locate the missed communities [25,26]. It is important to note that our results may be subject to selection bias as we purposefully selected upazilas for LQAS to ensure geographic variability, which is critical for the larger IR. In addition, there is a risk of selection bias during the LQAS, although to reduce this bias, we instructed our data collectors to begin data collection from the north-east corner of each selected EPI cluster.

Our experience highlights the potential challenge in reaching ZD and UI children in conflict-affected areas. We were unable to conduct the LQAS survey in Rangamati district due to the presence of various armed groups in the areas and resulting a travel ban enacted by the local administration. While support from key stakeholders, and their understanding of the need to assess and address ZD and UI in this area based on our secondary analysis, may facilitate our access to this district for the larger implementation research, this is not guaranteed.

The results of the determinant analysis suggest that while wealth does not exert much influence over a child being ZD or UI, mother’s educational status, access to media, and practice or ability to make ANC visits were most strongly associated with children’s vaccination status. The greater the status that a mother had in regards to these factors, the less likely was it for her child to be ZD or UI. This is consistent to studies in Eritrea and Nigeria that showed mothers with primary level and above education or mothers who made more ANC visits, were more likely to have children that were fully immunized [27,28]. In addition to factors related to both ZD and UI, mothers who reported that “their last child as unwanted” were more likely to have a ZD child. This is similar to findings of a study [29] from Ethiopia that showed a positive association between mother’s contraceptive use and the children having full immunization coverage. The nature of this relationship needs further exploration to understand its implications for addressing ZD.

We found routine data from the national HMIS to be useful for initial identification of ZD areas because it is one of the few sources that provides district and upazila level estimates and because it is publicly available. That said, the current system has a number of limitations that, if addressed, would make it more useful for identifying ZD areas. In particular, the currently available data only include rural areas which leaves out the 31% of the population that live in CCs and municipalities. Having coverage data for these areas, including at zonal level is critical to understanding the true situation and reaching the ZD and UI children who live there. Moreover, the routine data uses denominator estimates that in some cases result in illogical coverage estimates. Innovative approaches to address this denominator issue, which is a common challenge for using routine health information [30–32], require further exploration but should be a priority for the Directorate General Health Services (DGHS). Any efforts in this regard should account for geographic differences that may relate to denominator estimates (e.g., high levels of migration, quality of data). Improved denominators would assist in identifying ZD children.

Based on our qualitative analysis, key demand -side barriers that contribute towards children being ZD and UI include population migration, irrational fear of side-effects, busy schedules, and misconceptions and hesitancy, though the importance of these varied by area to some extent. The most prominent supply-side obstacle, and one that was mentioned in all areas, is the critical shortage of healthcare workers, especially HAs. This is exacerbated by (and contributes to) the limited opportunities to provide good client communications, EPI centers located too far from communities, and lack of transport options. Identification of ZD children is hindered by inaccurate denominators and population movements. Notably, our qualitative analysis along with evidence from quantitative analysis, enabled us to explore the hindrances and enabled us to few recommendations that may help policymakers and programme managers in identifying the ZD and UI areas and in designing of programmes to reduce the ZD and UI children in Bangladesh.

Based on our qualitative analysis, the key demand -side barriers that contribute towards children being ZD and UI include population migration, irrational fear of side-effects, busy schedules, misconceptions and hesitancy. We found critical shortage of healthcare workers, especially HAs, to be the most prominent supply-side obstacle in all study areas. This factor was exacerbated by (and further contributed to) limited opportunities to provide good client communications. Other prominent factors were EPI centers located quite distant from communities, and lack of transportation. Furthermore, inaccurate denominators and population movements hinder identification of ZD children. The demand and supply side barriers found to be similar for low coverage, drop-out, left out and ZD/UI [12,33–36], need to be addressed by the DGHS on priority basis. Notably, our qualitative analysis along with evidence from quantitative analysis, enabled us to explore the hindrances and enabled us to offer few recommendations that may help policymakers and programme managers in identifying the ZD and UI areas as well as in designing a programmes to reduce the ZD and UI children in Bangladesh.

In conclusion, our study shows that pockets of ZD and UI children do exist in Bangladesh despite the stellar performance of the country’s EPI programme and high levels of full immunization coverage. Children who are ZD or UI need to be reached so that they can also benefit from life-saving vaccines. This paper provides the methods and approaches with which the ZD and UI children as well as the missed communities were identified and explores the demand- and supply-side barriers that lead to children becoming ZD or UI within the country. These methods are both effective and easily replicable identify ZD and UI children. The drivers of ZD and UI along with the barriers to immunization provide potential areas for intervention by policy-makers and can inform interventions to be tested in future implementation research.

## Supporting information

S1 Table Socio-economic determinants for children being ZD [BDHS 2017–18]. (DOCX)

S2 Table Socio-economic determinants for children being UI [BDHS 2017–18]. (DOCX)

S1 Dataset LQAS data. (XLSX)

S2 Dataset DHIS2 2022. (XLSX)

S3 Dataset EPI CES data (2014–2019). (XLSX)
