# Supplementary material for: Identifying the zero-dose and under-immunized children in Bangladesh: Approaches and experiences
Source: PLoS One. 2024 Oct 28;19(10):e0312171. doi: 10.1371/journal.pone.0312171 (PMC11515957; doi:10.1371/journal.pone.0312171)
Supplement: S1 Table — (DOCX) [file pone.0312171.s001.docx]

**Supporting Information**

**Supplementary Tables**

**S1 Table.** Socio-economic determinants for children being ZD [BDHS 2017-18]

| **Covariates** | **n** | **Crude** | | **Adjusted** | |
| --- | --- | --- | --- | --- | --- |
|  |  | **OR (95.0% CI)** | **p-value** | **OR (95.0% CI)** | **p-value** |
| Sex of child | | | | | |
| Male (Ref) | 2356 | - | - | - | - |
| Female | 2148 | 1.48 (1.035-2.117) | 0.032 | 1.402 (0.954-2.062) | 0.086 |
| Number of ANC visits |  |  |  |  |  |
| 0 (Ref) | 365 | - | - | - | - |
| 1-3 | 1941 | 0.392 (0.245-0.628) | <0.001 | 0.531 (0.319-0.882) | 0.015 |
| >=4 | 2198 | 0.179 (0.106-0.304) | <0.001 | 0.374 (0.205-0.683) | 0.001 |
| Division | | | | | |
| Rangpur (Ref) | 501 | - | - | - | - |
| Barisal | 474 | 2.933 (0.928-9.272) | 0.067 | 1.821 (0.564-5.879) | 0.316 |
| Chattogram | 744 | 1.987 (0.637-6.195) | 0.237 | 1.21 (0.369-3.966) | 0.753 |
| Dhaka | 678 | 3.171 (1.06-9.479) | 0.039 | 1.931 (0.622-5.992) | 0.255 |
| Khulna | 465 | 2.45 (0.75-8.009) | 0.138 | 2.235 (0.669-7.463) | 0.191 |
| Mymensingh | 546 | 3.492 (1.151-10.59) | 0.027 | 2.527 (0.822-7.772) | 0.106 |
| Rajshahi | 479 | 1.58 (0.443-5.633) | 0.481 | 0.95 (0.233-3.871) | 0.943 |
| Sylhet | 617 | 10.72 (3.853-29.853) | <0.001 | 5.024 (1.741-14.493) | 0.003 |
| Type of residence | | | | | |
| Urban (Ref) | 1560 | - | - | - | - |
| Rural | 2944 | 0.975 (0.673-1.414) | 0.895 | 0.774 (0.497-1.204) | 0.255 |
| Mothers educational attainment | | | | | |
| No education (Ref) | 278 | - | - | - | - |
| Primary incomplete | 764 | 0.785 (0.459-1.344) | 0.378 | 0.986 (0.548-1.774) | 0.961 |
| Primary complete | 470 | 0.614 (0.329-1.144) | 0.124 | 0.617 (0.311-1.225) | 0.168 |
| Secondary incomplete | 1931 | 0.205 (0.116-0.364) | <0.001 | 0.262 (0.134-0.513) | <0.001 |
| Secondary complete or higher | 1061 | 0.125 (0.058-0.268) | <0.001 | 0.221 (0.089-0.547) | 0.001 |
| Wealth quintile | | | | | |
| Poorest (Ref) | 953 | - | - | - | - |
| Poorer | 922 | 0.686 (0.41-1.148) | 0.151 | 0.888 (0.505-1.563) | 0.682 |
| Middle | 799 | 0.84 (0.504-1.399) | 0.503 | 1.525 (0.837-2.779) | 0.168 |
| Richer | 895 | 0.809 (0.491-1.332) | 0.404 | 1.47 (0.765-2.822) | 0.247 |
| Richest | 935 | 0.272 (0.134-0.549) | <0.001 | 0.659 (0.264-1.645) | 0.371 |
| Wanted last child | | | | | |
| Wanted then (Ref) | 3545 | - | - | - | - |
| Wanted later | 598 | 1.422 (0.892-2.266) | 0.139 | 1.710 (1.026-2.85) | 0.040 |
| Wanted no more | 361 | 1.464 (0.825-2.598) | 0.192 | 1.212 (0.661-2.22) | 0.535 |
| Mother’s occupation | | | | | |
| Working (Ref) | 1815 | - | - | - | - |
| Not working | 2689 | 2.879 (1.838-4.508) | <0.001 | 2.797 (1.732-4.518) | <0.001 |
| Media access | | | | | |
| Yes (Ref) | 2457 | - | - | - | - |
| No | 2047 | 2.409 (1.656-3.504) | <0.001 | 1.493 (0.941-2.369) | 0.089 |
