# Supplementary material for: Identifying the zero-dose and under-immunized children in Bangladesh: Approaches and experiences
Source: PLoS One. 2024 Oct 28;19(10):e0312171. doi: 10.1371/journal.pone.0312171 (PMC11515957; doi:10.1371/journal.pone.0312171)
Supplement: S2 Table — (DOCX) [file pone.0312171.s002.docx]

**Supporting Information**

**Supplementary Tables**

**S2 Table.** Socio-economic determinants for children being UI [BDHS 2017-18]

| **Covariates** | **n** | **Crude** | | **Adjusted** | |
| --- | --- | --- | --- | --- | --- |
|  |  | **OR (95.0% CI)** | **p-value** | **OR (95.0% CI)** | **p-value** |
| Sex of child | | | | | |
| Male (Ref) | 2192 | - | - | - | - |
| Female | 2000 | 1.196 (0.956-1.495) | 0.117 | 1.184 (0.936-1.498) | 0.158 |
| Number of ANC visits | | | | | |
| 0 (Ref) | 335 | - | - | - | - |
| 1-3 | 1805 | 0.509 (0.366-0.708) | <0.001 | 0.644 (0.454-0.913) | 0.013 |
| >=4 | 2052 | 0.261 (0.183-0.371) | <0.001 | 0.45 (0.303-0.667) | <0.001 |
| Division | | | | | |
| Rangpur (Ref) | 474 | - | - | - | - |
| Barisal | 431 | 1.33 (0.724-2.442) | 0.358 | 1.038 (0.557-1.935) | 0.906 |
| Chattogram | 698 | 1.925 (1.14-3.251) | 0.014 | 1.632 (0.943-2.824) | 0.080 |
| Dhaka | 634 | 1.844 (1.08-3.149) | 0.025 | 1.631 (0.924-2.878) | 0.092 |
| Khulna | 436 | 2.105 (1.203-3.684) | 0.009 | 2.196 (1.229-3.923) | 0.008 |
| Mymensingh | 501 | 1.954 (1.126-3.393) | 0.017 | 1.575 (0.896-2.77) | 0.115 |
| Rajshahi | 451 | 1.448 (0.801-2.62) | 0.221 | 1.316 (0.712-2.432) | 0.381 |
| Sylhet | 567 | 3.563 (2.152-5.899) | <0.001 | 2.403 (1.408-4.102) | 0.001 |
| Type of residence | | | | | |
| Urban (Ref) | 1450 | - | - | - | - |
| Rural | 2742 | 1.151 (0.905-1.463) | 0.252 | 0.891 (0.675-1.177) | 0.416 |
| Mothers educational attainment | | | | | |
| No education (Ref) | 257 | - | - | - | - |
| Primary incomplete | 711 | 0.717 (0.49-1.049) | 0.086 | 0.852 (0.568-1.279) | 0.439 |
| Primary complete | 434 | 0.509 (0.325-0.796) | 0.003 | 0.57 (0.354-0.919) | 0.021 |
| Secondary incomplete | 1813 | 0.334 (0.232-0.481) | <0.001 | 0.439 (0.291-0.664) | <0.001 |
| Secondary complete or higher | 977 | 0.187 (0.118-0.295) | <0.001 | 0.323 (0.19-0.548) | <0.001 |
| Wealth quintile | | | | | |
| Poorest (Ref) | 894 | - | - | - | - |
| Poorer | 846 | 0.71 (0.516-0.978) | 0.036 | 0.851 (0.603-1.202) | 0.36 |
| Middle | 738 | 0.696 (0.497-0.975) | 0.035 | 0.981 (0.666-1.446) | 0.923 |
| Richer | 843 | 0.741 (0.538-1.019) | 0.065 | 1.153 (0.773-1.719) | 0.486 |
| Richest | 871 | 0.329 (0.221-0.492) | <0.001 | 0.606 (0.357-1.03) | 0.064 |
| Wanted last child | | | | | |
| Wanted then (Ref) | 3298 | - | - | - | - |
| Wanted later | 560 | 0.942 (0.678-1.309) | 0.724 | 0.987 (0.691-1.41) | 0.942 |
| Wanted no more | 334 | 1.204 (0.824-1.759) | 0.338 | 0.961 (0.643-1.437) | 0.848 |
| Mother’s occupation | | | | | |
| Working (Ref) | 1725 | - | - | - | - |
| Not working | 2467 | 1.33 (1.052-1.681) | 0.017 | 1.416 (1.097-1.827) | 0.007 |
| Media access | | | | | |
| Yes (Ref) | 2295 | - | - | - | - |
| No | 1897 | 1.983 (1.578-2.493) | <0.001 | 1.401 (1.056-1.858) | 0.019 |
